# Supplementary material for: Painting with light-powered bacteria
Source: Nat Commun. 2018 Feb 22;9:768. doi: 10.1038/s41467-018-03161-8 (PMC5823856; doi:10.1038/s41467-018-03161-8)
Supplement: Supplementary file 1 — Supplementary Information [file 41467_2018_3161_MOESM1_ESM.pdf]

Supplementary information for 'Painting with light–powered bacteria'

Jochen Arlt, Vincent A Martinez, Angela Dawson, Teuta Pilizota, & Wilson C K Poon

22nd January 2018

Supplementary Figures

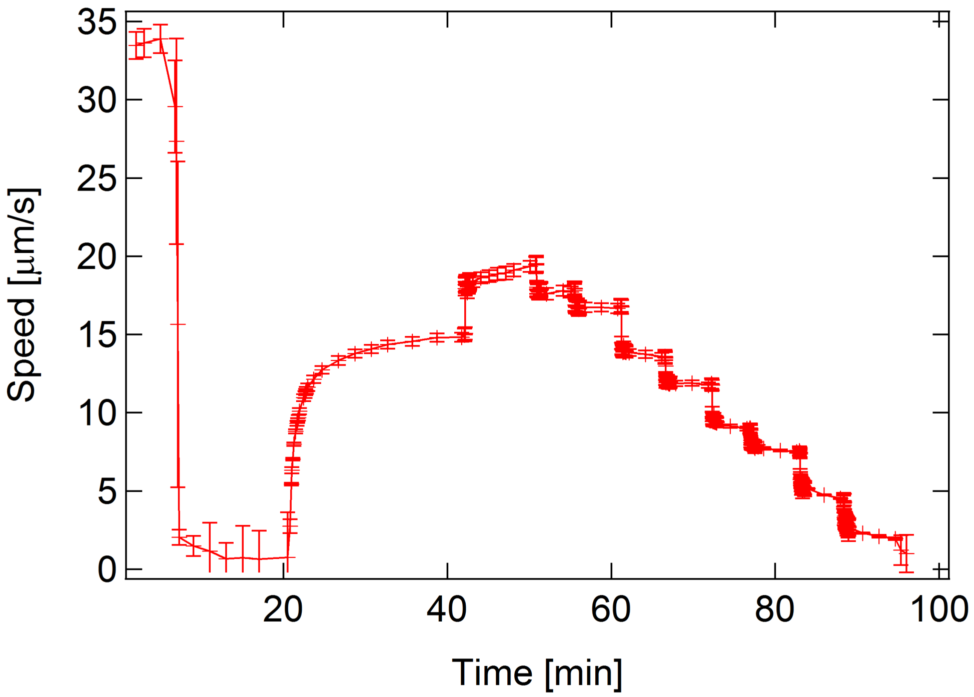

Supplementary Figure 1: **Swimming speed of AD10** (sample OD = 1) during oxygen depletion and for different light intensities: Within ≈ 7 min of sealing the sample oxygen was depleted and swimming ceased. Green light was switched on at ≈ 20 min. Illumination intensity was changed at various points throughout this experiment to provide data for the speed *vs.* intensity plot in the main manuscript (inset of Fig. 1a). Error bars are standard deviations of the q averages of the DDM speed $v(q)$.

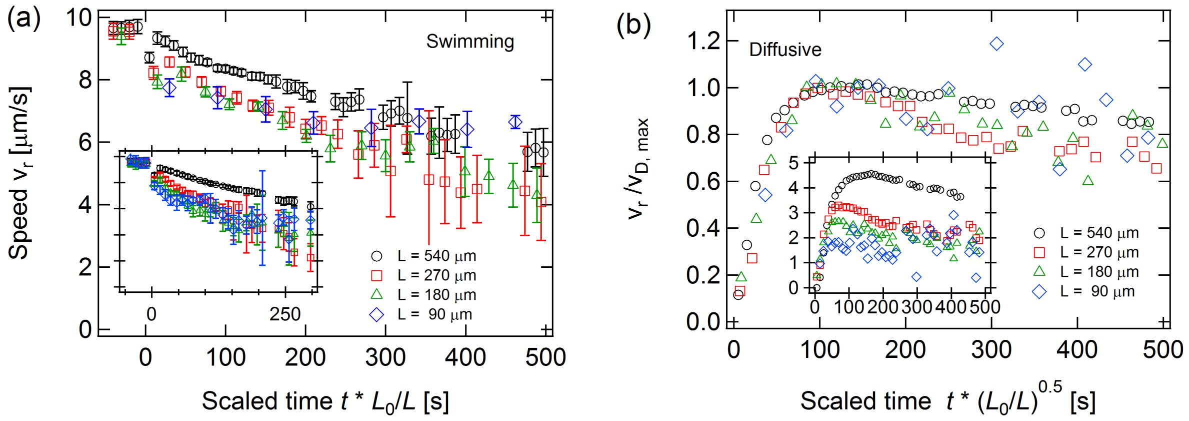

Supplementary Figure 2: **Average swimming speed inside emptying squares** of various sizes L when starting with swimming or diffusive cells. For swimming cells (a) the speed $\bar{v}_r$ does not quite stay constant at $v_{\rm sat} = 9.5\,\mu\rm m\,s^{-1}$ but drops about linearly with time. For diffusive cells (b) the speed displays more complex dynamics: $\bar{v}_r$ initially increases linearly with time before approaching a maximum value which increases with the size of the square L. Scaling speed with $\bar{v}_{\rm D,max}$ and time with $\sqrt{L/L_0}$ (with $L_0 = 540\mu m$) leads to good data collapse. (Insets: raw speed data against time without any scaling). Error bars are standard deviations of the q averages of the DDM relative density $\rho(q)$.

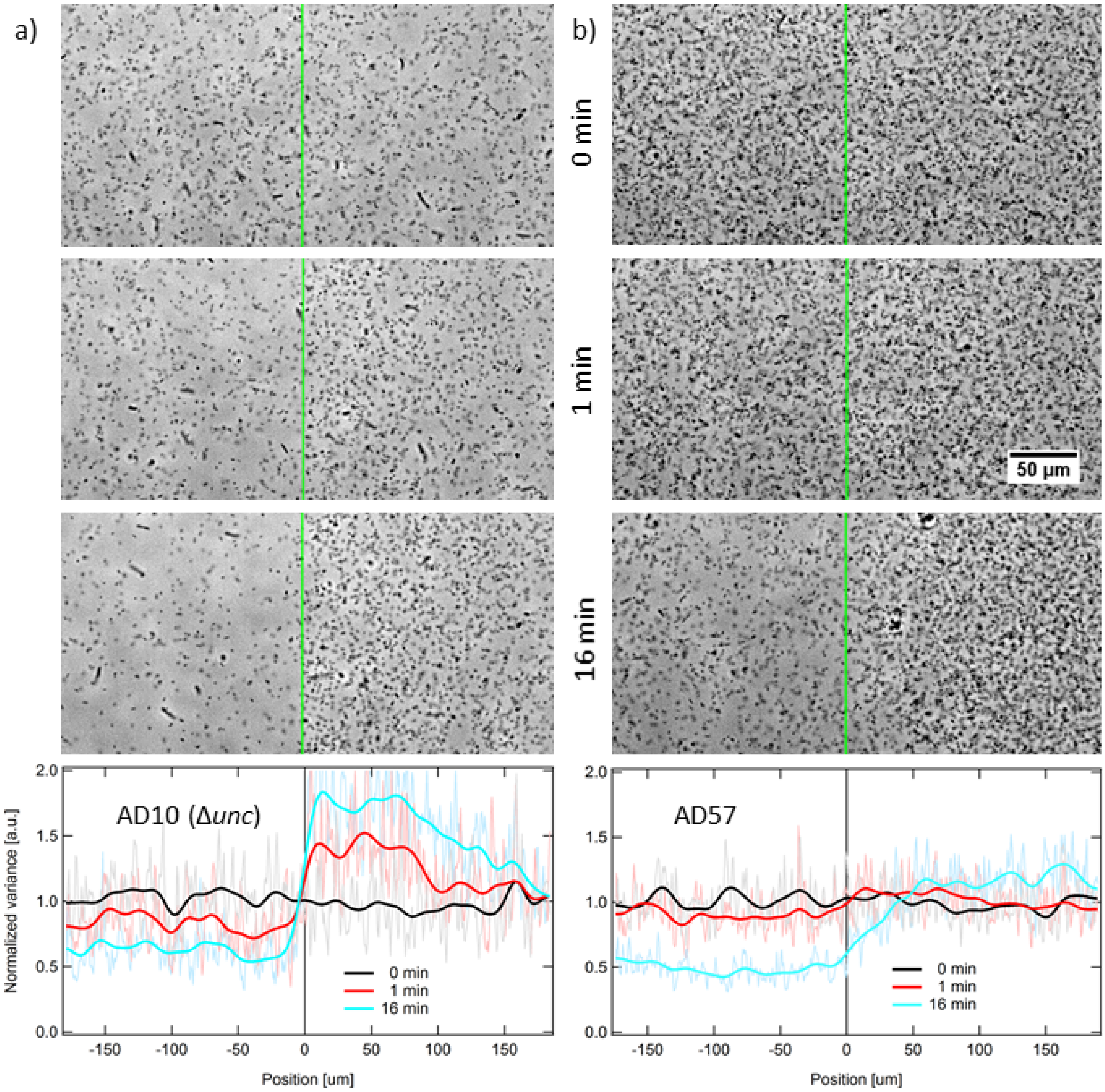

Supplementary Figure 3: **Spatial control** of a) AD10 strain compared to b) AD57 strain. Phase contrast images (Nikon PF 20x/0.5 objective) of the samples just before (0 min) as well as 1 min and 16 min after blocking the green light on the right half of the image (boundary position indicated by green lines). The corresponding variance profiles (normalised by the mean variance under uniform illumination) are shown underneath the images. The numerically smoothing (bold lines; using a binomial smoothing filter with $n = 201$) of the raw data (faint lines) highlights the underlying change in cell density. Recalling that the variance measures the cell density, we find that for the AD57 strain the density difference develops much more slowly and shows a much more gradual transition, making it less suitable for STASA.

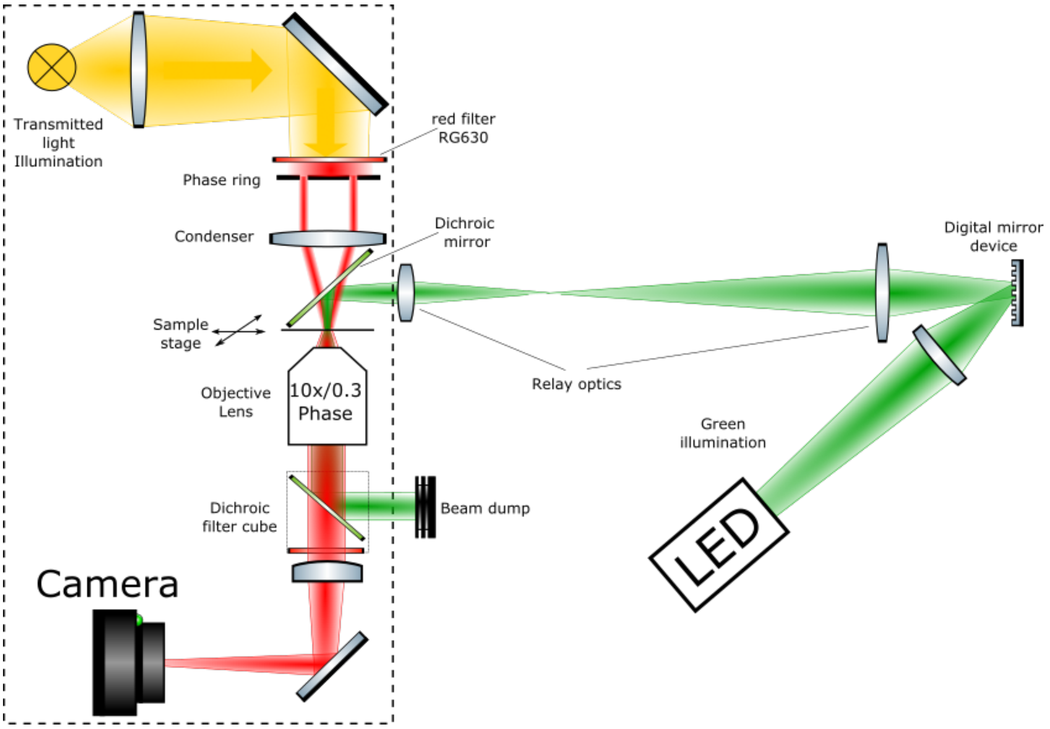

Supplementary Figure 4: **Schematic of the optical setup.** A standard inverted research microscope (Nikon TE2000, indicated by the black dashed box) is used for low magnification phase contrast imaging of our samples (Nikon PF 10x/0.3 Ph1 objective). A red filter (RG630, Schott Glass) in the illumination path ensures the light for imaging can not power the swimming of our bacteria. A standard epi-fluorescence filter cube (Nikon G-2A) only lets this red light reach the camera (MC 1462, Mikrotron). The spatial pattern is generated using a computer controlled digital mirror device (DMD) illuminated by LED light (SOLA SE II, Lumencor) filtered to a green wavelength range (510–560 nm). The DMD is conjugate with the sample plane and therefore any pattern on the DMD is imaged onto the sample using a demagnifying set of relay lenses. The green light is introduced onto the sample using a 'trans' geometry by inserting a dichroic mirror in between the long working distance condenser and the sample. This gives a large illuminated area while retaining phase contrast imaging capability.

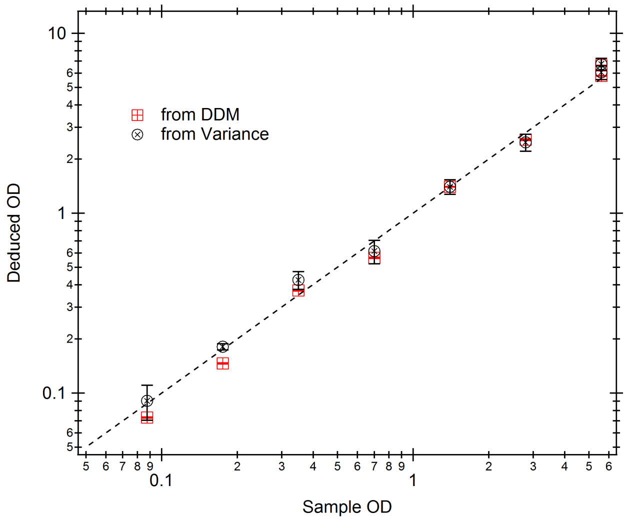

Supplementary Figure 5: **Cell density** deduced from DDM and intensity variance measurements (see Methods) for a series of 7 samples starting at a measured optical density of OD = 5.6 and diluted successively by factors of 2. Both analysis methods were applied to the same movies and yield consistent results in quantitative agreement with the prepared sample densities throughout the measured range. Both data sets have been calibrated by assuming the data point for OD 1.4 was measured correctly. Error bars are standard deviations from the mean.

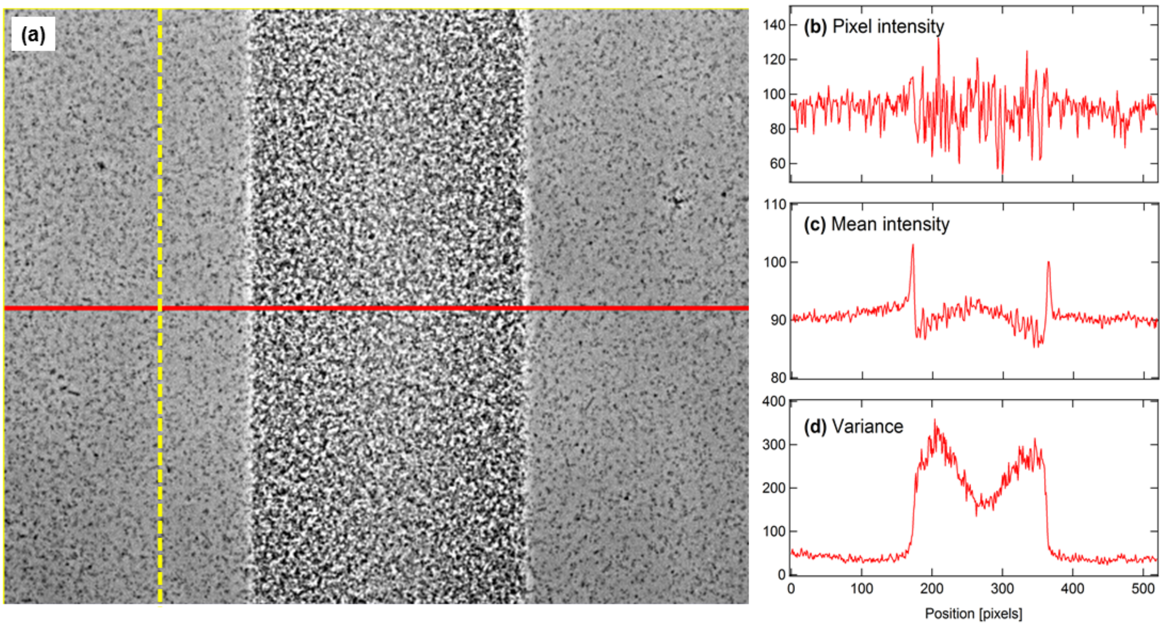

Supplementary Figure 6: **Extracting density profiles** from phase contrast images: (a) phase contrast image with a line (red) used for the pixel intensity profile, one vertical line (dashed yellow) used for calculating the mean intensity and variance at this horizontal position. Intensity profile of (b) single pixel line and the (c) averaged profile, which is dominated by the bright halo artifacts next to the edges. (d) Intensity variance profile.

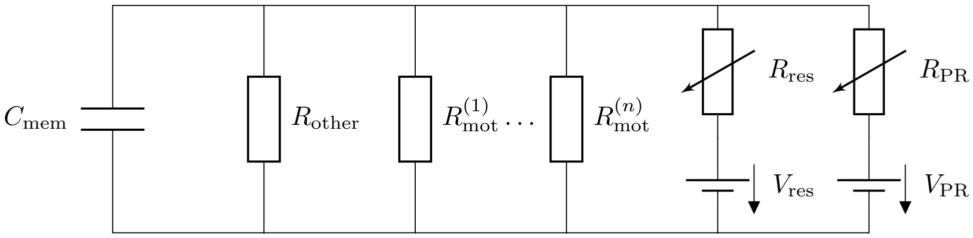

Supplementary Figure 7: **Equivalent circuit** of the bacterial PMF generation and dissipation: $R_{\text{mot}}^{(i)}$ = resistance of individual flagella motors in parallel with R_{other} = combined resistance of all other membrane channels, C_{mem} = membrane capacitance, V_{res} = PMF generated by respiratory enzymes, R_{res} = variable resistor modelling the effect of oxygen concentration on respiratory enzymes, V_{PR} = proteorhodopsin-generated PMF, R_{PR} = variable resistor modelling the effect of light on PR. (Redrawn after [1].)

Supplementary Note 1 Circuit model

The effect of proteorhodopsin (PR) on the proton motive force (PMF) can be modelled using a simple equivalent circuit, Supplementary Fig. 7 [1]. We use this model for our experiments by interpreting $R_{\rm res}$ as the effect of oxygen concentration on the respiratory circuit: $R_{\rm res} \to \infty$ when oxygen is exhausted. The effect of light on PR is modelled as a variable resistor, $R_{\rm PR}$, which Walter *et al.* have argued to be related to the incident light intensity $\mathcal{I}$ by $R_{\rm PR}(\mathcal{I}) = R_{\rm PR}^{\infty}(\mathcal{I} + \mathcal{K})/\mathcal{I}$ (with $\mathcal{K}$ a constant). Using the assumption that the average swimming speed is proportional to PMF, this leads to a similar intensity dependence of the speed $\bar{v}_{\rm sat}(\mathcal{I}) = \bar{v}_{\max}\mathcal{I}/(\mathcal{I} + \mathcal{I}_{1/2})$ under complete oxygen starvation. Fitting this to our experimental data gives a maximum swimming speed $\bar{v}_{\max} = 28.4(7)\,\mu\text{m}\,\text{s}^{-1}$ and an intensity to reach half this speed of $\mathcal{I}_{1/2} = 10.7(4)\,\text{mW}\,\text{cm}^{-2}$, suggesting that our PR is significantly more efficient than the one used by Walter *et al.* (for which $\mathcal{I}_{1/2} = 60\,\text{mW}\,\text{cm}^{-2}$) [1].

We suggest that the stopping time is controlled by the discharge of the PMF through an equivalent resistor-capacitor circuit, $\tau_{\rm off} = R_{\rm tot}C_{\rm mem}$. Here $C_{\rm mem}$ is the equivalent capacitance of the membrane, and the total equivalent resistance $R_{\rm tot}$ is made up of the equivalent resistance of the motors ($R^{(i)}_{\rm mot}$) and all other membrane channels ($R_{\rm other}$) connected in parallel. The specific capacitance of bacterial membranes is $\approx 0.6\,\mu\text{F}\,\text{cm}^{-2}$ [2]. A $2\,\mu\text{m} \times 1\,\mu\text{m}$ spherocylindrical *E. coli* cell has $2\pi\,\mu\text{m}^2$ surface area, giving $C_{\rm mem} \approx 4 \times 10^{-14}\text{F}$. We have previously estimated [3] that the proton current through a motor is $\approx 2 \times 10^4\ \text{H}^+\ \text{s}^{-1}$, which is driven by a PMF of $\approx -150\,\text{mV}$, giving a resistance of $\approx 0.5 \times 10^{14}\Omega$ per motor. On average, each cell has, say, ≈ 5 motors. These constitute resistors in parallel, so that all together, $R_{\rm mot} \approx 0.1 \times 10^{14}\Omega$. The effective resistors constituted by the membranes, cell wall and other channels ($R_{\rm other}$) are connected in parallel with $R_{\rm mot}$, so that $R_{\rm tot} < R_{\rm mot}$, possibly by an order of magnitude or more. Thus, $\tau_{\rm off} \sim R_{\rm tot}C_{\rm mem} < 1\,\text{s}$, as claimed in the main text.

Supplementary Note 2 Swimming speed inside illuminated squares

In the main text we reported measurements of how long it took to empty an illuminated square (side L) of cells, starting from swimming or stationary cells. The emptying time scales as L and $L^{1/2}$ respectively. This is the scaling expected for random ballistic swimmers leaving a box of size L. This is appropriate for our experiments, as the persistence length of our smooth swimming bacteria is comparable to the box size. Here we report the average speed of cells remaining in the illuminated square, $\bar{v}_{\rm r}$, as a function of time in the two cases. Starting from swimming cells, our simplistic model presented in the main manuscript assumed a constant swimmming speed $\bar{v}_{\rm r} = \bar{v}_{\rm sat}$, whereas the actually measured speed $\bar{v}_{\rm r}$ decreased with time, Supplementary Fig. 2(a): our bacteria have a broad swimming speed distribution and the fastest-swimming cells leave the square first, leading to a change in $P(v)$ and a corresponding drop in the mean speed. This drop becomes more rapid the smaller the square, displaying approximately the same scaling with L as the average cell density shown in the main text.

Starting from stationary (meaning diffusing) cells, $\bar{v}_{\rm r}(t)$ is non-monotonic, Supplementary Fig. 2(b). Immediately after illumination inside the square is switched on, cells accelerate over a time scale of $\sim \tau_{\rm on}$; later, $\bar{v}_{\rm r}$ decreases with time for the same reason as in the case of starting with swimming cells: the fastest cells leave earlier. The maximum speed reached by a swimmer depends on the time it spent in the box, leading to $v_{\rm D,max} = a\text{t}_{\rm D} \sim \sqrt{L}$, based on $\text{t}_{\rm D} \sim \sqrt{L}$ as demonstrated in the main text. This is in agreement with the measured population averaged $\bar{v}_{\rm D,max} \sim \sqrt{L}$ shown in Fig. 3c of the main manuscript, and the data collapse of $\bar{v}_{\rm r}/\bar{v}_{\rm D,max}$ with scaled time $t(L_0/L)^{0.5}$ as shown in Supplementary Fig. 2(b).

Supplementary Note 3 STASA for AD10 vs AD57

We demonstrate the effect of $\tau_{\rm stop}$ on STASA by projecting a bright-dark intensity step onto cells that had been uniformly illuminated for $\approx 10\,\text{min}$ (so that the population had reached saturation speed, $\bar{v}_{\rm AD10} = 17.5\,\mu\text{m}\,\text{s}^{-1}$ and $\bar{v}_{\rm AD57} = 11.8\,\mu\text{m}\,\text{s}^{-1}$).

Supplementary Fig. 3 shows snapshots of samples of a) the fast-stopping AD10 strain and b) the AD57 strain just before (0 min) as well as 1 min and 16 min after introducing the intensity step. For AD10 a clear difference

in cell density between two sharply defined halves is apparent very quickly, with cells entering the dark region stopping almost instantaneously. Cells in the illuminated half keep on swimming until they enter a dark region, leading to a drop in cell number in the illuminated region and an accumulation of cells just inside the dark region. For AD57 the cells in the dark half initially only slow down but keep on swimming at a lower speed for several minutes. Therefore there is initially very little effect on the cell density, and only after a much longer time a far more gradual pattern emerges, as demonstrated by the profiles of the intensity variance (reproduced from the main text in Supplementary Fig. 3).

Supplementary References

[1] Walter, J. M., Greenfield, D., Bustamante, C. & Liphardt, J. Light-powering *Escherichia coli* with proteorhodopsin. *Proc. Natl. Acad. Sci. USA* **104**, 2408–12 (2007).

[2] Bai, W., Zhao, K. & Asami, K. Dielectric properties of *E. coli* cell as simulated by the three-shell spheroidal model. *Biophys. Chem.* **122**, 136 – 142 (2006).

[3] Schwarz-Linek, J. *et al.* *Escherichia coli* as a model active colloid: A practical introduction. *Colloids Surf. B* **137**, 2–16 (2016).
