# Supplementary material for: Painting with light-powered bacteria
Source: Nat Commun. 2018 Feb 22;9:768. doi: 10.1038/s41467-018-03161-8 (PMC5823856; doi:10.1038/s41467-018-03161-8)
Supplement: Supplementary file 3 — Description of Additional Supplementary Files [file 41467_2018_3161_MOESM3_ESM.pdf]

Description of Additional Supplementary Files

File Name: Supplementary Movie 1
Description: 40s movie showing the initial formation of the `UoE' pattern. This corresponds to the runup to fig. 2a in the manuscript. One pixel corresponds to 1.4µm and the frame rate has been reduced from the 100 fps of the original movie to 2 fps and is played back at 5× the real speed.

File Name: Supplementary Movie 2
Description: Movie showing initial 40s just after the inversion of the `UoE' pattern. This corresponds to the transition between fig 2c and d in the manuscript. Frame-rate adjusted as for movie M1.

File Name: Supplementary Movie 3
Description: Timelapse image series of the `UoE' pattern being formed and inverted. This illumination pattern is switched on at time `0:00' when the sample is still uniform (corresponding to movie 1) and becomes clearer over time. After 9min the pattern is inverted (corresponding to movie M2) and again becomes more pronounced over time. The outline of the letter `o' is marked to highlight the switching of the boundary as the pattern is inverted.

File Name: Supplementary Movie 4
Description: Timelapse image series showing the mouth of the smiley pattern being flipped horizontally and then `erasing' the whole pattern by illuminating the sample with a uniform light field.
